# Matrix metalloproteinases operate redundantly in Arabidopsis immunity against necrotrophic and biotrophic fungal pathogens

**DOI:** 10.1371/journal.pone.0183577

**Published:** 2017-08-23

**Authors:** Puyan Zhao, Fei Zhang, Dilin Liu, Jafargholi Imani, Gregor Langen, Karl-Heinz Kogel

**Affiliations:** Institute of Phytopathology, Justus Liebig University Giessen, Heinrich-Buff-Ring, Giessen, Germany; University of Nebraska-Lincoln, UNITED STATES

## Abstract

Matrix metalloproteinases (MMPs) are evolutionarily conserved and multifunctional effector molecules playing pivotal roles in development and homeostasis. In this study we explored the involvement of the five *Arabidopsis thaliana* At-MMPs in plant defence against microbial pathogens. Expression of *At2-MMP* was most responsive to inoculation with fungi and a bacterial pathogen followed by *At3-MMP* and *At5-MMP*, while *At1-MMP* and *At4-MMP* were non-responsive to these biotic stresses. Loss-of-function mutants for all tested At-MMPs displayed increased susceptibility to the necrotrophic fungus *Botrytis cinerea* and double mutant *at2*,*3-mmp* and triple mutant *at2*,*3*,*5-mmp* plants developed even stronger symptoms. Consistent with this, transgenic Arabidopsis plants that expressed *At2-MMP* constitutively under the Cauliflower mosaic virus 35S promoter showed enhanced resistance to the necrotrophic pathogen. Similarly, resistance to the biotrophic Arabidopsis powdery mildew fungus *Golovinomyces orontii* was also compromised particularly in the *at2*,*3-mmp* / *at2*,*3*,*5-mmp* multiplex mutants, and increased in *At2-*MMP overexpressor plants. The degree of disease resistance of *at-mmp* mutants and *At2-*MMP overexpressor plants also correlated positively with the degree of MAMP-triggered callose deposition in response to the bacterial flagellin peptide flg22, suggesting that matrix metalloproteinases contribute to pattern-triggered immunity (PTI) in interactions of Arabidopsis with necrotrophic and biotrophic pathogens.

## Introduction

Matrix metalloproteinases (MMPs) are a group of conserved eukaryotic endoproteinases belonging to the metzincin superfamily that contain a zinc ion in the active site. Structural features of MMP proteins include a signal peptide, a propeptide and a catalytic domain. The propeptide contains the conserved sequence PRCGXPD termed “cysteine switch”. The catalytic domain is characterized by the peptide sequence HEXGHXXGXXH with the three histidine residues responsible for coordinating with the catalytic zinc ion, followed by a conserved methionine forming the so-called Met-turn [1–3]. MMPs are secreted or anchored to the cell surface, thereby restricting their catalytic activity to membrane proteins and proteins in the secretory pathway or extracellular space [3]. Notably, characterised vertebrate MMPs can degrade numerous extracellular substrates, including virtually all extracellular matrix proteins.

Vertebrate MMPs have distinct and partially overlapping functions and substrate specificities [4–5]. Their physiological functions are linked to embryonic development, tissue remodeling, angiogenesis, immunity, and wound healing. Imbalanced MMP activities are associated with many pathological processes and destructive diseases such as tumor progression, rheumatism, and osteoarthritis [4–7]. The number of MMPs identified in insects is remarkably lower when compared with mammals. For example, 24 homologs are known from humans, while only two have been found in the fruit fly *Drosophila melanogaster* [8] and three in the red flour beetle *Tribolium castaneum* [9]. Yet, recent findings provide evidence that the physiological roles of insect MMPs expanded beyond those related to development to also include functions in immunity [9–10].

Despite of their prevalence in the plant kingdom, knowledge about plant MMPs is vague. They are encoded by intronless genes, display the common features mentioned above and play versatile roles in growth, development and immunity [1]. Plant MMPs are phylogenetically close to the human MMP7. In 1991, the first plant MMP, soybean metalloendoproteinase-1 (SMEP1), was described and it participates in tissue remodelling during leaf expansion [11–13]. Subsequently, cucumber *Cs1-MMP* was suggested to be involved in senescence-related programmed cell death [14]. *MtMMPL1* from the legume *Medicago truncatula* functions as a negative regulator of *Rhizobium* infection. Its overexpression led to reduced nodule formation, while its knock-down supported the establishment of the symbiosis [15]. Due to the presence of a point mutation in the active site of the catalytic domain of MtMMPL1, it remains unclear whether the protease activity is compromised *in vivo* and whether the active protease site is essential for the functionality of MtMMPL1. Soybean *GmMMP2* is responsive to the oomycete pathogen *Phytophthora sojae*, both in compatible and incompatible interactions, and to the bacterial pathogen *Pseudomonas syringae* pv. *glycinea* [16]. Similarly, expression of *NMMP1* in *Nicotiana benthamiana* and *NtMMP1* in tobacco was induced in response to bacterial pathogens while silencing in *N*. *benthamiana* led to enhanced susceptibility against *P*. *syringae* pv. *tabaci* [17–18]. Recently, tomato *SlMMPs* were reported to be involved in cell death control or resistance against the pathogens *Botrytis cinerea* and *Pseudomonas syringae* pv. *tomato* [19, 20]. These reports indicate that MMPs from various plant species are involved in defence against microbial infections.

In Arabidopsis, the MMP family consists of five members named *At1-MMP* to *At5-MMP* [21]. Their encoded proteins are predicted to be located in the plasma membrane or apoplast, though subcellular localization has not yet been experimentally verified. A previous study on At2-MMP suggested a function in senescence and flowering as an *at2-mmp* mutant exhibited early senescence and late flowering [22]. Analysis of microarray data available from Genevestigator also suggests that expression of some *At-MMPs* is induced upon pathogen attack or treatment with microbe-associated molecular patterns (MAMPs) such as flg22 [23–24].

In the present study we investigated the five Arabidopsis MMPs during microbial infection. We show that *At-MMPs* are responsive to microbial pathogens with diverse life styles, and provide first evidence for the regulatory role of distinct At-MMPs in plant innate immunity.

## Materials and methods

### Plant materials and growth conditions

The Arabidopsis materials included the accession Col-0 (wild-type, WT), *at1-mmp* (GK-575B01.01), *at2-mmp* (GK-416E03.01), *at3-mmp* (SM_3.28404), *at5-mmp* (SALK_119909), as well as signaling mutants *ICS1* (SALK_042603.55.70.x), *npr1-3* (CS3802), *jar1-1*(CS8072), *npr1-1* (CS3726) and *ein2-1* (CS3071). The surface sterilized seeds were germinated in Petri dishes containing 1/2 Murashige-Skoog (MS) medium (Sigma-Aldrich, St. Louis, MO, USA) with 1% sucrose and 0.7% agar under 8/16 h (day/night) conditions in a growth chamber (fluorescent cool white, 180 μmol/m^2^ s^1^ photon flux density, 25^°^C) for 10 days. Seedlings were transferred to soil (soil: sand = 3:1 [v/v]; Fruhstorfer Erde Typ T, Hawita, Lauterbach, Germany) and grown under short-day condition (8h light/16h darkness regime, 22°C/18°C and 60% humidity).

### Production and identification of *at-mmp* double and triple mutants

The mutant *at2*,*3-mmp* was generated by crossing of single mutants *at2-mmp* and *at3-mmp*. Resulting seeds were propagated and DNA was extracted from leaves and used for PCR with primers (see S1 Table) to identify homozygous mutants in F_2_ generation. For production of the *at2*,*3*,*5-mmp* mutant, the single mutant *at5-mmp* and double mutant *at2*,*3-mmp* were used for crossing and seeds of their F2 progeny were used to identify the homozygous line with primers described in S1 Table.

### Generation of *At2-MMPox* over-expressor plants

For over-expression of *At2-MMP* (AGI: AT1G70170), genomic DNA of Arabidopsis (WT) was used to amplify the full length coding sequence by PCR using primer pairs At2-MMP2-FL-F and At2-MMP2-FL-R (see S1 Table). The restriction sites *Bam*HI and *Hin*dIII were introduced in the gene specific full-length primers. After cloning into pGEMT easy vector (Promega, Madison, USA) and sequencing, the fragment was released through *Bam*HI/*Hin*dIII digestion, ligated into the plasmid p35S-Nos-BM (DNA Cloning Service, Hamburg, Germany), containing CaMV 35S promoter, resulting in p35S-MMP2-Nos. The expression cassette was subcloned into the *Sfi*I sites of pLH6000 binary vector. The plasmids pLH6000-p35S-MMP2 and pLH6000-p35S (empty vector control) were used to transform *Agrobacterium tumefaciens* strain AGL1. Stable transgenic plants were produced by floral dip [25]. Seedlings from T_0_ plants were selected on 1/2 MS containing 30 mg/l hygromycin (Duchefa Biochemie, Haarlem, The Netherlands).

### Pathogen inoculation and quantification

For *Botrytis cinerea* strain B05.10 inoculations, Arabidopsis leaves from four-week-old plants were detached and placed in Petri dishes containing 0.5% agar medium. Inoculation was performed by placing 5 μl of conidia suspension on the leaf center (5x10^4^ conidia ml^-1^ in 12 g/l potato dextrose broth, PDB; Duchefa Biochemie, Haarlem, The Netherlands). Disease symptoms were evaluated at 3 to 6 dpi by determining lesion size using Image J software [26]. For gene expression analysis, 2x10^5^ conidia/ml were used for inoculation of Arabidopsis plants and PDB was used as mock. After inoculation, the plants were placed in a transparent box tightly covered to keep humidity high. The boxes were incubated in a growth chamber under short-day condition. Leaf samples were harvested at 0 h, 8 h, 16 h, 24 h, 48 h and 72 h after inoculation for gene expression analysis.

For *Pseudomonas syringae* pv *tomato* DC3000 (*Pst*) inoculation, bacteria were streaked out from a –80°C glycerol stock onto a plate of King’s B medium (1% protease peptone, 0.15% anhydrous K_2_HPO_4_, 1.5% glycerol, 1.5% agar, pH 7.0) containing 50 mg/l rifampicin and grown at 28°C. After two days, the bacteria were scrapped off with sterile 10 mM MgCl_2_ using a glass spatula and the optical density was adjusted to OD_600_ = 0.1. One half of leaves were infiltrated with a one ml needleless syringe, containing the bacterial suspension or mock-infiltrated with MgCl_2_. The non-injected halves of leaves were harvested for gene expression assay.

For powdery mildew *Golovinomyces orontii* inoculation, four-week-old healthy plants were sprayed with 20 to 40×10^3^ conidia/ml spore suspension. At 11 dpi, the conidia were rinsed from leaves and the number of conidia per mg of fresh weight was determined by microscopy [27].

A culture of *Serendipita (*syn: *Piriformospora*) *indica* (DSM11827) was maintained at 22°C on complex medium (CM) [28]. Chlamydospores from three-week-old *S*. *indica* plates were collected in 0.02% sterilized Tween20-H_2_O and adjusted to 5×10^5^ chlamydospores/ml. For each square Petri plate (12 cm x 12 cm), 1 ml spore suspension was distributed on three-week-old Arabidopsis roots followed by 30 sec gentle shaking to ensure uniform distribution of the spores. The mock treatment was done with 0.02% Tween20-H_2_O. Roots were harvested at 0, 1, 3 and 7 dpi and flash frozen in liquid nitrogen. All root samples were stored at -80°C prior to RNA extraction.

### DNA/RNA extraction and gene expression analysis by RT-PCR

For identification of T-DNA insertion mutants and transgenic plants, genomic DNA was extracted from Arabidopsis leaves as described [22] For gene expression analysis, total RNA was extracted using TRIzol (Invitrogen, Karlsruhe, Germany) from leaves at the indicated time points after *B*. *cinerea* and *P*. *syringae* inoculation, and cDNA synthesis was performed with a cDNA synthesis kit (Fermentas, St. Leon-Rot, Germany). The resulted cDNA was used as template for RT-PCR. The expression of *At-MMPs* was compared to the expression of the reference gene Arabidopsis *Ubiquitin* 5 (*UBQ5*, AGI: AT3G62250). Twenty-eight cycles were used for the amplification of *UBQ5* and 35 cycles were used for the amplification of *At-MMPs*. Primer pairs are listed in S1 Table.

### MAMP-induced callose deposition

The callose assay was performed as described [29]. Briefly, five-week-old leaves were infiltrated with 1 μM flg22 for 24 h. Leaves were cut, fixed and destained in ethanol: glacial acetic acid (3:1) with 1x change of the solution until the leaves were transparent. Leaves were re-hydrated in 70% ethanol for 15 min, then in 50% ethanol for 15 min. After several washes with water, leaves were incubated for 1 to 2 h in 150 mM K_2_HPO_4_ (pH 9.5) containing 0.01% aniline blue. Callose deposits were detected using a Zeiss Axioplan2 microscope (excitation, 365 nm; emission, 420 nm), and quantified from digital photographs with Image J software [26].

### Production of recombinant At2-MMP and enzyme analysis

For generation of recombinant At2-MMP protein, a construct containing only the catalytic domain corresponding to the predicted mature MMP2 (Mat-MMP2) was prepared. The fragment was amplified by PCR from pGEMT-At2-MMP and cloned in-frame into pET32a(+) (Novagen, Madison, USA) containing Thioredoxin-6xHis-S tag at the N terminus. Recombinant protein was produced and purified in *Escherichia coli* strain BL21(DE3) pLysS cells following standard procedure as described [21]. Briefly, protein expression was induced by isopropyl-β-D-thiogalactopyranoside (IPTG, 1 mM). After 4 h induction at 37°C, *E*.*coli* cells were harvested and lysed using lysis buffer (10 mM Tris-Hcl pH 8.0, 1u/ml DNaseI, 1 mg/ml lysozyme, 0.1mM PMSF). Though majority of Mat-MMP2 protein existed as inclusion body after three times sonication, a small portion of Mat-MMP2 was found in the supernatant after the third and fourth sonication and they were tested for enzymatic activity.

Enzyme analysis was performed as described [21]. Degradation of myelin basic protein (MBP) was assayed using bovine MBP (Sigma-Aldrich, St. Louis, MO, USA) at a final concentration of 0.25 μg/μl in 100 mM Tris–HCl pH 7.5, 5 mM CaCl_2_, 0.05% Brij 35, and 0.02% NaN_3_. Recombinant Mat-MMP2 (0.1 μM) was incubated with MBP in a 15 μl reaction volume for the indicated time (30 min) at 37°C, and products were analyzed by 20% SDS-PAGE to visualize MBP degradation. The gels were stained with Coomassie Blue overnight and then bleached for 1 h for photography.

### Proteolytic activity of intercellular washing fluid (IWF)

Intercellular washing fluid (IWF) was extracted from Arabidopsis WT, EV and *At2-MMPox* plants as described [30]. Myelin basic protein (MBP) was used as substrate to determine the proteolytic activity of the IWF. IWF from different plants was incubated with MBP for 10 h. The products were separated by 20% SDS-PAGE. Inhibition of metalloprotease activity was determined by the addition of EDTA at a concentration ranging from 100 nM to 5 mM.

### Subcellular localization of At2-MMP-GFP fusion protein

The GFP coding sequence without stop codon was amplified from 35S-GFP using primers GFP-*Sal*I and GFP-*Xho*I (S1 Table) and cloned in frame into 35S-At2-MMP at its endogenous *Sal*I site. For transient transformation, leaves were detached from five-week-old WT plants. The leaves were co-bombarded with the construct 35S-At2-MMP-GFP and a control plasmid 35S-mCherry. At 24 h after bombardment, the subcellular localization of MMP2-GFP was analyzed by confocal laser scanning microscopy (Leica, Bensheim, Germany). Plasmolysis was performed with 50% glycerol for 5 min.

### Statistical analysis

Results are expressed as means ± standard deviation (SD) or ± standard error (SE) as indicated in the figure legends and represent at least three biological repetitions. Statistical analysis was performed using Student’s t-Test. P<0.05 was considered to be significant.

## Results

### *At2-MMP* and *At3-MMP* are responsive to fungal and bacterial pathogens

**Fig 1:**
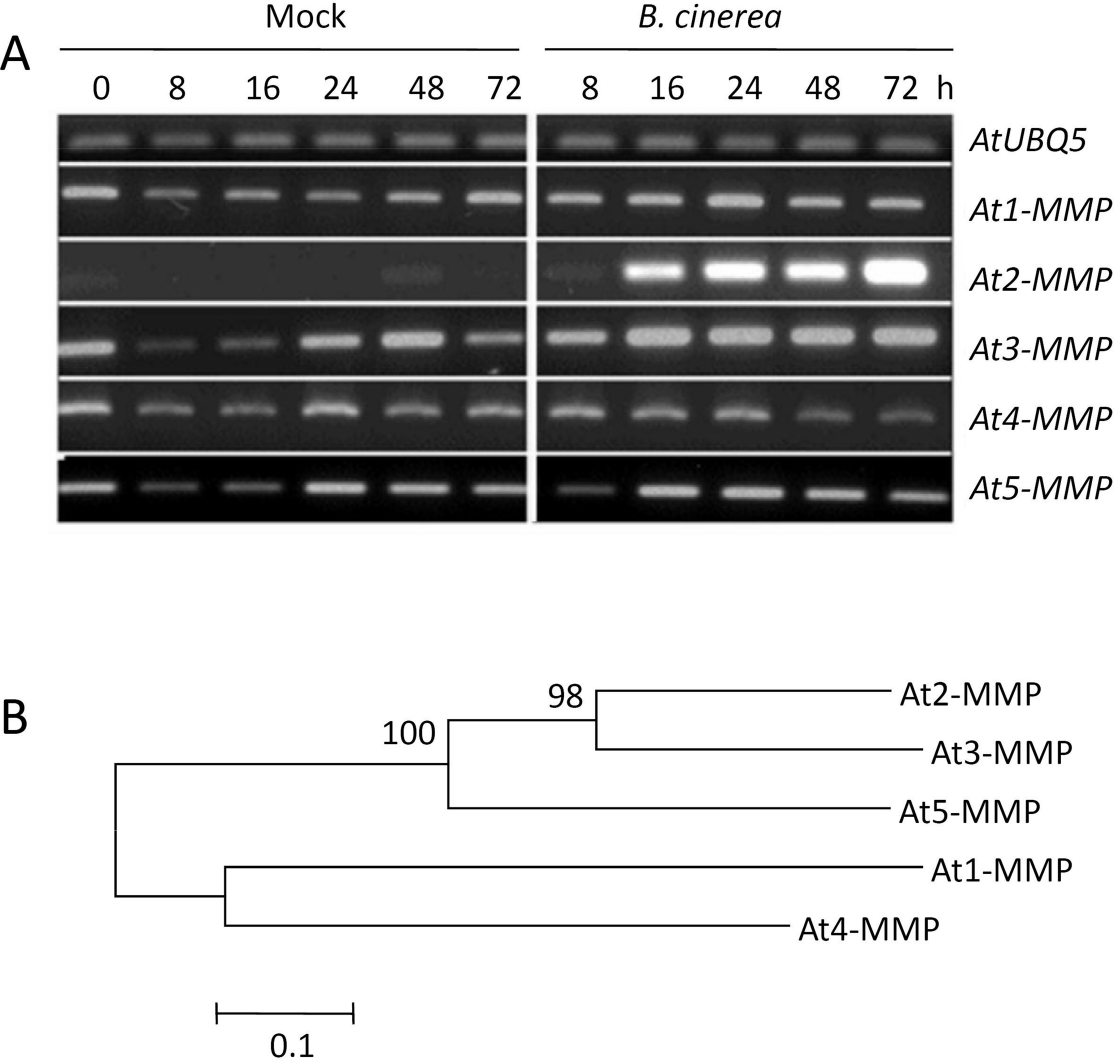
Responses of *At-MMPs* upon *Botrytis cinerea* infection. (A) Expression profiles of *At-MMPs* in Arabidopsis accession Col-0 (WT) leaves in response to *Botrytis cinerea*. Five-week-old plants were inoculated with the fungus by placing 5 μl of spore suspension (2x10^5^ spores/ml in potato dextrose broth [PDB]) on the center of the rosette leaves. Mock treatment was performed with PDB. Total RNA was extracted from leaves at the indicated time points and used for RT-PCR. UBQ5 was used as an internal control. Experiments were independently repeated three times with similar results. (B) Molecular phylogenetic analysis of MMPs in Arabidopsis. The evolutionary history was inferred by Neighbor-Joining method using a conserved region spanning the cysteine switch and Met-turn motif of AtMMP protein sequences.

As several plant MMPs were shown to be induced by pathogens [16–17], we addressed the question whether the five established Arabidopsis MMPs are responsive to microbial infection. To this end, leaves from five-week-old Arabidopsis WT plants were inoculated with 2×10^5^ conidiospores/ml of the necrotrophic ascomycete *Botrytis cinerea*, the causal agent of gray mold in many plant species. We found a slight downregulation of all MMPs during darkness (8h and 16h time points) compared to ubiquitin, probably caused by the circadian rhythm (Fig 1). *At2-MMP* was strongly induced by fungal infection from 16 hours post inoculation (hpi) onwards, while it showed the lowest basal expression level of all *At-MMPs*. *At3-MMP* was induced starting at an early time point (8 hpi) and *At5-MMP* showed a transient induction at 16 hpi, though both genes showed rather high expression in non-inoculated leaves. In contrast, expression of *At1-MMP* and *At4-MMP* was not induced by the fungus.

To extend this analysis, we further assessed expression of *At-MMPs* after inoculation with biotrophic microbes. Expression of *At2-MMP* and *At3-MMP* was induced after inoculation of Arabidopsis (WT) leaves with the virulent bacterial pathogen *Pseudomonas syringae* pv. *tomato* DC3000 (*Pst*). *At5-MMP* was transiently expressed at 4 hpi. Consistently, *At1-MMP* and *At4-MMP* did not show a change in expression (S1A Fig). In contrast, powdery mildew infection with a virulent strain of the obligate biotrophic fungus *Golovinomyces orontii* did not substantially induce *At-MMPs* within a three-day-time period (S1B Fig). We further assessed whether *At-MMPs* are induced by a beneficial root endophyte. To this end, Arabidopsis (WT) roots were inoculated with the fungus *Serendipita indica* and *At-MMPs* expression was measured at 24, 72, and 168 hpi. We found that all the five *At-MMPs* were not induced by the fungus (S2 Fig).

### Induction of *At2-MMP* and *At3-MMP* by *B*. *cinerea* is independent of salicylate, jasmonate, and ethylene

**Fig 2:**
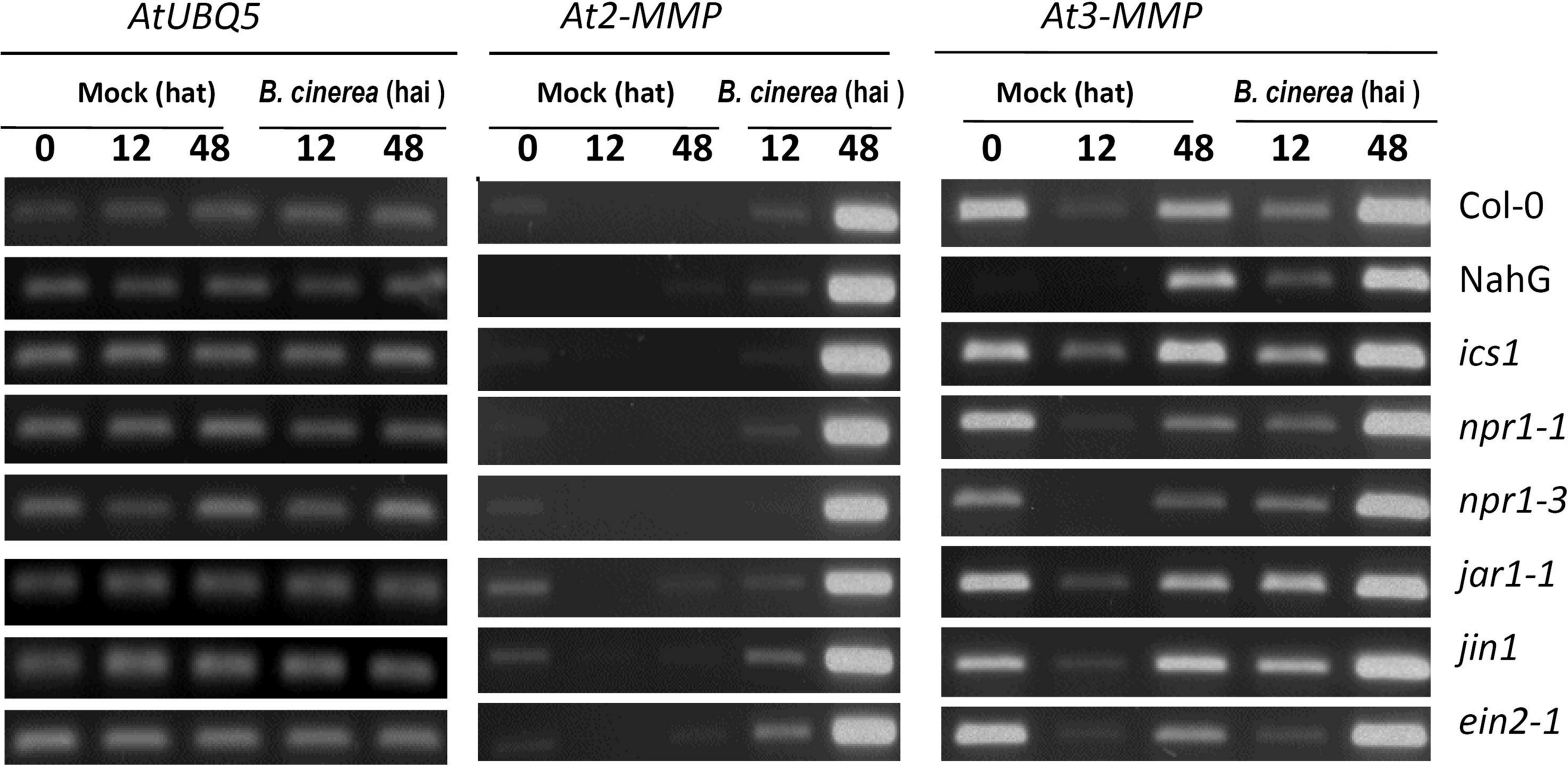
Expression profiles of *At2-MMP* and *At3-MMP* in hormone signaling mutants after *B*. *cinerea* infection. Five-week-old Arabidopsis WT plants, SA pathway mutants (*NahG*, *ics1*, *npr1-3*, JA pathway mutants (*jar1-1*, *jin1*, *npr1-1*) and ethylene pathway mutant (*ein2-1*) were inoculated with *B*. *cinerea* by placing 5 μl spore suspension (2x10^5^ spores/ml in PDB) or PDB (mock) on the center of the rosette leaves. Total RNA was extracted from leaves at the indicated time points and used for RT-PCR. UBQ5 was used as an internal control. Experiments were independently repeated three times with similar results. hat: hours after treatment, hai: hours after inoculation.

To determine whether *B*. *cinerea*-induced expression of *At-MMPs* is affected by compromised defence signaling, expression of the two pathogen-responsive genes *At2-MMP* and *At3-MMP* was assessed in Arabidopsis mutants affected in SA signaling (*NahG*, SA-degrading SA hydroxylase; *ics1*, *isochorismate synthase*; *npr1-3*, *non-expressor of PR proteins 1–3*), JA signaling (*jar1-1*, *jasmonate-responsive 1–1*; *jin1*, *jasmonate-insensitive 1; npr1-1*, *non-expressor of PR proteins 1–1*), and ethylene signaling (*ein2-1*, *ethylene insensiti*ve 2–1). In all the tested mutants, the induction pattern of *At2-MMP* and *At3-MMP* was unaffected and similar to WT plants (Fig 2).

### Domain structure and subcellular localization of At2-MMP

**Fig 3:**
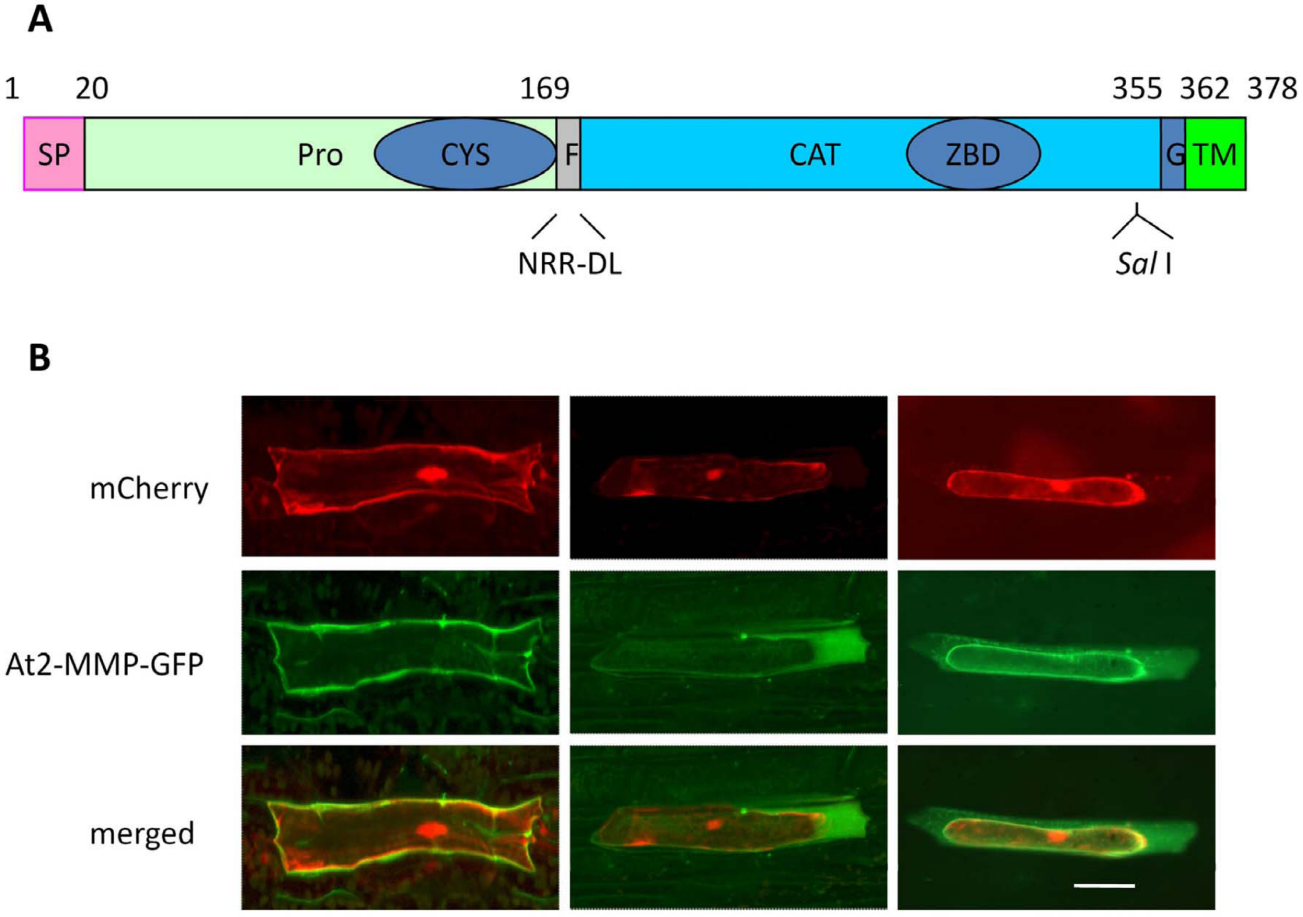
Structure and subcellular localization of At2-MMP. (**A**) Domain structure of an At2-MMP-GFP fusion construct. Numbers denote the position of amino acids (aa). SP: signal peptide; Pro: propeptide domain; CYS: cysteine switch; F: predicted furin-cleavage site; CAT: catalytic domain; ZBD: zinc-binding domain; G: putative GPI-anchor modification site; TM: transmembrane domain. The *Sal*I site (GTCGAC, aa 337–338) is downstream of the zinc-binding domain and upstream of the GPI-anchor modification site. The GFP coding sequence was integrated at the *Sal*I site. (**B**) Subcellular localization of At2-MMP upon transiently expression of 35S::At2-MMP2-GFP together with 35S::mCherry in Arabidopsis WT leaves. At2-MMP-GFP was detected 24 h after bombardment. Left panel: without plasmolysis; middle and right panel: five min after plasmolysis induced with 50% glycerol. Bar = 20 μm.

Since *At2-MMP* showed the strongest response to pathogen infection, we further analyzed the cellular location of At2-MMP. Arabidopsis *At2-MMP* (AGI: AT1G70170, NP_177174) encodes a protein of 378 amino acids (aa) with a predicted molecular mass of 42 kD and an isoelectric point of 5.78 for the full-length preproprotein. It has a predicted signal peptide (aa 1–20) and a putative GPI-anchor modification site followed by a transmembrane domain at the C-terminus (Fig 3A). An extra cysteine residue is present in the propeptide that is predicted to form a disulfide bridge with the cysteine residue in the “cysteine switch” motif, thus may not be operational as was found for NtMMP1 by Mandal et al [31]. At2-MMP is predicted to be located in the extracellular space by SubLoc and TargetP [32–33]. However, WoLF PSORT [34] prediction suggests that At2-MMP is bound to the plasma membrane. To analyse the location, we constructed a *GFP* fusion of *At2-MMP* under control of the 35S cauliflower mosaic virus promoter with the GFP coding sequence inserted into the endogenous unique *Sal*I site of *At2-MMP* (Fig 3A), which locates between the zinc-binding domain and the putative GPI-anchor modification site. The fusion construct 35S-At2-MMP-GFP was delivered into Arabidopsis (WT) leaves through particle bombardment. As shown in Fig 3B (middle-left panel), transiently produced At2-MMP-GFP was exclusively localized at the cell periphery. By contrast, the mCherry signal from a co-bombarded 35S-mCherry plasmid was found throughout the cell periphery, cytoplasm and nucleus (Fig 3B, top-left panel). To assess whether At2-MMP2-GFP was bound to the plasma membrane or secreted into the extracellular space, we induced plasmolysis with 50% glycerol. The GFP signal was mainly observed in the space between plasma membrane and cell wall (Fig 3B, centre panel) and associated with the plasma membrane (Fig 3B, middle-right panel), while the mCherry signal remaining within the cytoplasm (Fig 3B, top-middle and top-right panel). We concluded that At2-MMP is present both at the plasma membrane and in the apoplast.

### At-MMPs contribute to resistance to necrotrophic *B*. *cinerea*

**Fig 4:**
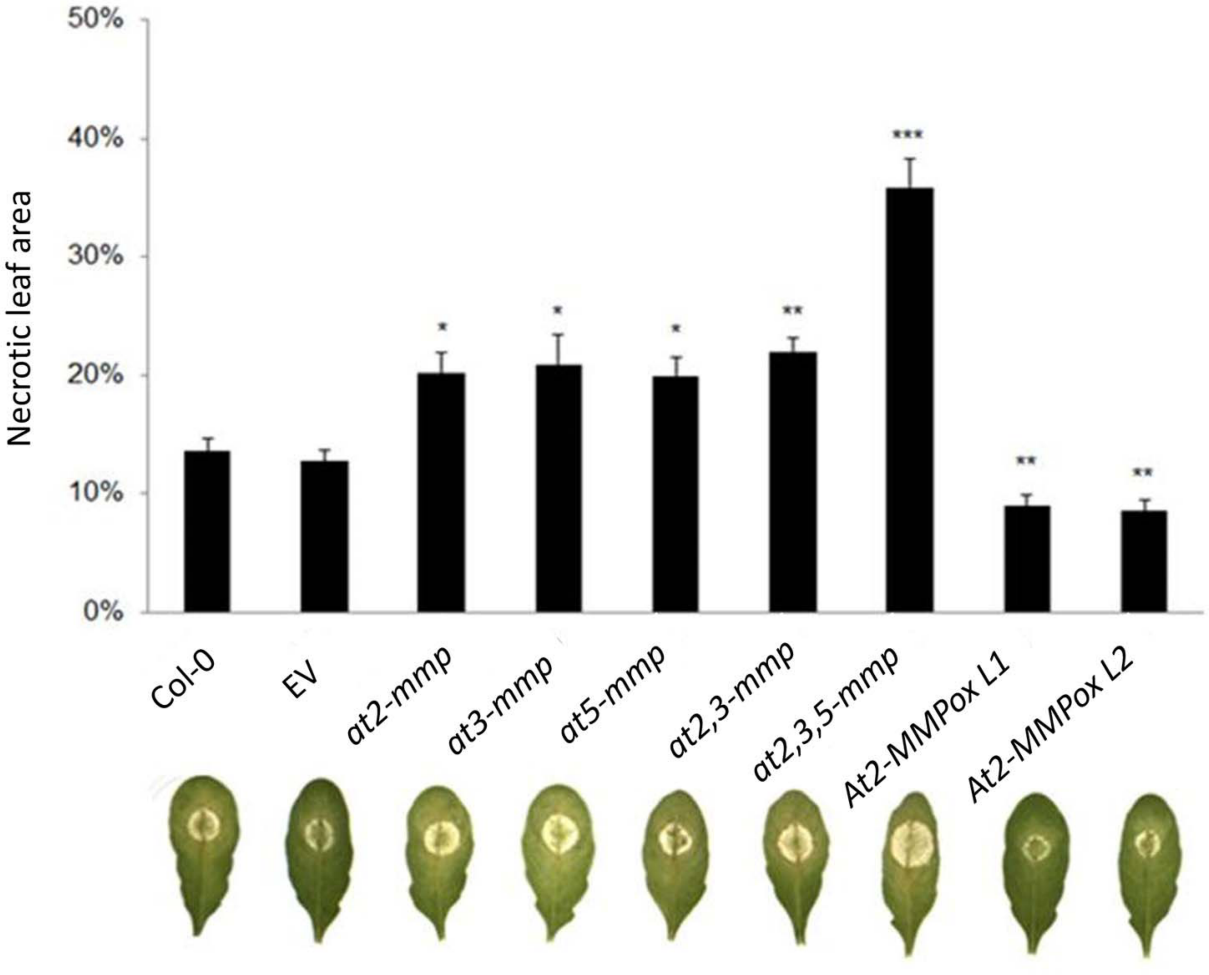
At2-MMP, At3-MMP, and At5-MMP are involved in basal resistance to *Botrytis cinerea*. Detached leaves from 4-week-old Arabidopsis plants were inoculated with fungal conidia. Five μl spore suspension adjusted to 50,000 conidia per ml were placed on the middle vein. Disease symptoms were evaluated at 4 dpi. The image shows representative symptoms out of 12 leaves per line. Experiments were repeated three times with similar results. Shown is the mean ± SE of one experiment. Significant differences are marked: *p < 0.05, **p < 0.01, ***p < 0.001 (Student’s *t*-Test).

We further assessed the involvement of pathogen-responsive *At-MMPs* in disease resistance using loss-of-function mutants. To this end, we tested the homozygous Arabidopsis mutants *at2-mmp*, *at3-mmp* and *at5-mmp* as well as the double mutant *at2*,*3-mmp* and the triple mutant at*2*,*3*,*5-mmp* that were obtained by crossing. All the confirmed homozygous mutant lines showed no difference in growth or morphology as compared to WT plants in the vegetative stage (not shown). Detached leaves of four-week-old plants were inoculated with *B*. *cinerea* conidia. We found that all tested *at-mmp* mutants were hyper-susceptible compared to WT plants (Fig 4). *at2*,*3*,*5-mmp* showed the highest rate of infection followed by *at2*,*3-mmp* and the single mutants *at2-mmp*, *at3-mmp* and *at5-mmp* as evidenced by their lesion sizes. Consistent with this, overexpression (ox) of *At2-MMP* rendered plants more resistant to *B*. *cinerea* as *At2-MMPox* lines L1 and L2 showed a greatly reduced lesion size compared with WT and transgenic empty vector (EV) control plants. As for the mutants, the *At2-MMPox* plants displayed otherwise no altered phenotype during vegetative growth. The data suggest that At2-MMP, At3-MMP and At5-MMP associate with disease resistance to necrotrophic *B*. *cinerea*. Moreover, consistent with the previously reported late flowering phenotype of *at2-mmp* plants [22], flowering started more early in *At2-MMP*ox lines compared to the WT plants (S3 Fig).

### At-MMPs are involved in basal resistance to biotrophic *G*. *orontii*

**Fig 5:**
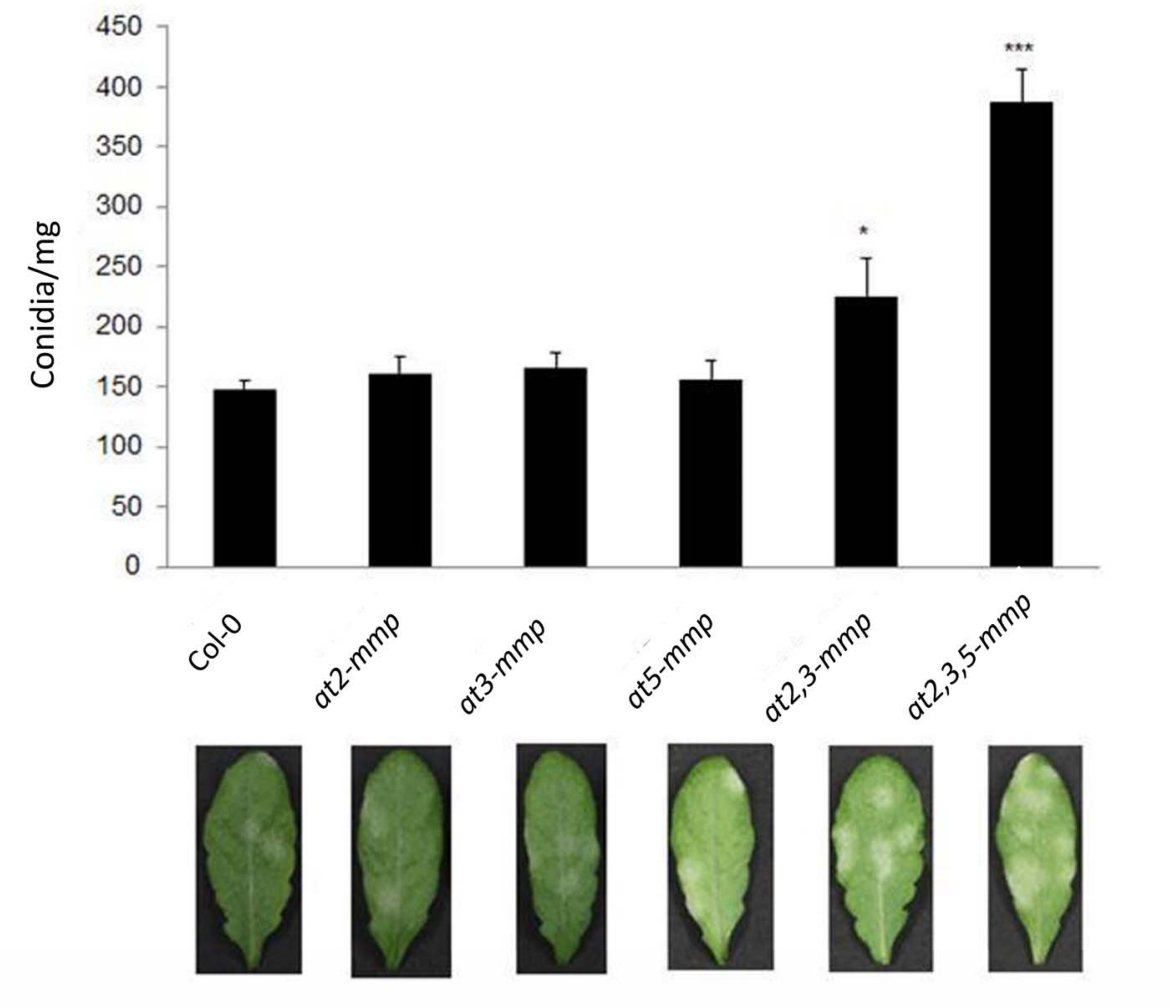
Disease symptoms in Arabidopsis WT plants and *at-mmp* mutants infected by the powdery mildew fungus *Golovinomycis orontii*. Four-week-old plants were spray-inoculated with 20–40×10^3^ conidia per ml suspension. Leaves were detached and photographed at 11 dpi. The amount of conidia per mg of leaf fresh weight was determined. At least 10 individually plants were treated in each line. The error bars indicate the standard error. Experiments were repeated three times with similar results. Significant differences are marked: *p < 0.05, ***p < 0.001 (Student’s t-Test).

We further assessed whether At-MMPs are also required for resistance to the biotrophic powdery mildew fungus *G*. *orontii*, though expression of *At-MMPs* was not substantially altered by infection. To this end, four-week-old Arabidopsis plants were inoculated with 20–40×10^3^ conidia/ml spore suspension, and the amount of newly formed conidia per mg of leaf fresh weight was quantified at 11 days post inoculation (dpi). Single *at-mmp* mutants did not show significant higher infections compared to WT. However, fungal infection increased strongly in *at2*,*3-mmp* (30%) and particularly in *at2*,*3*,*5-mmp* (110%) as compared with WT plants (Fig 5; S4 Fig). Taken together, these data show that the loss of function of several MMPs compromise basal plant resistance to the biotrophic powdery mildew fungus, indicating redundant function at least for the pathogen-responsive *At2-MMP*, *At3-MMP*, and *At5-MMP*.

### At-MMPs modulate flg22-triggered callose deposition

**Fig 6:**
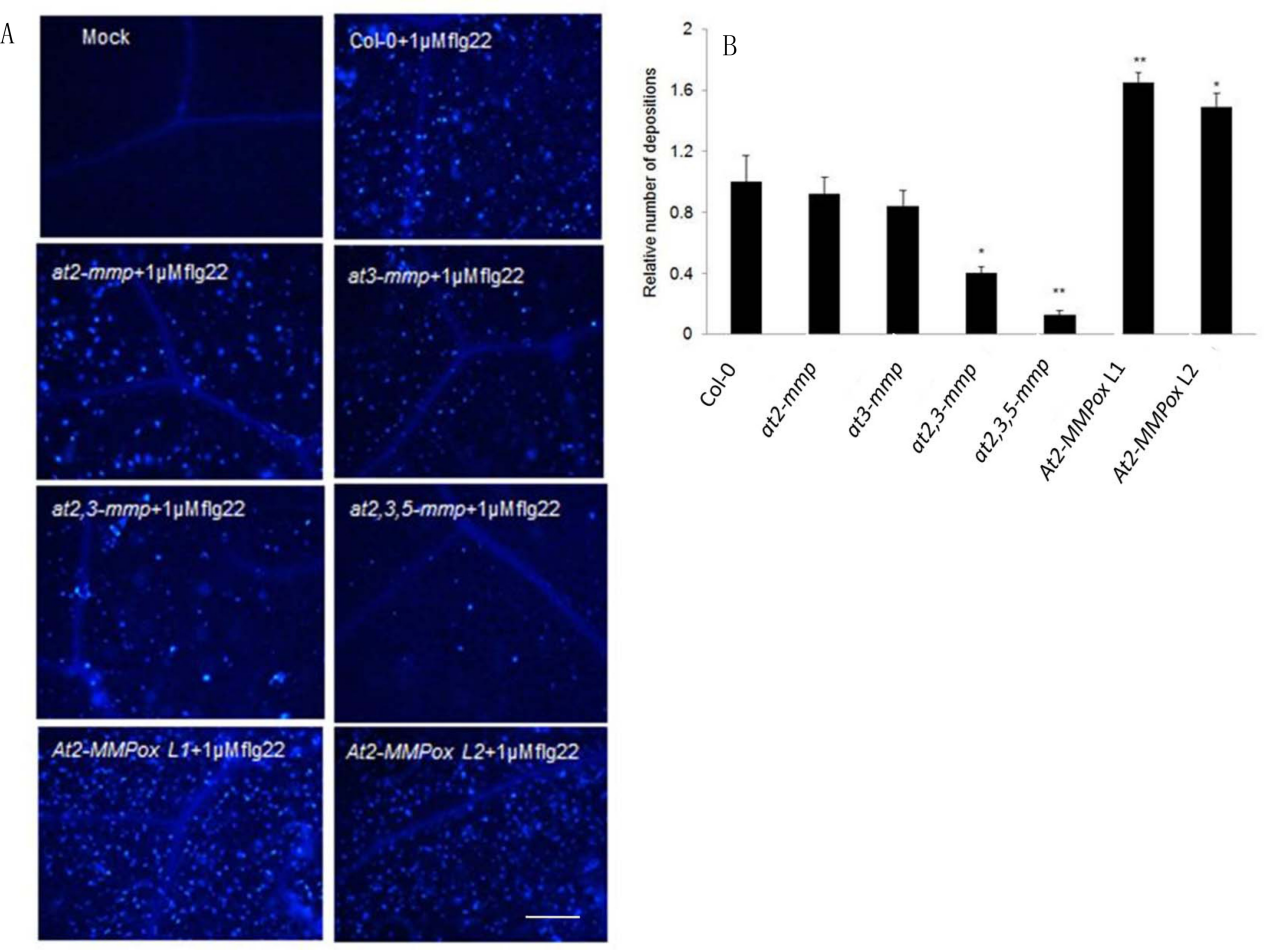
Immune responses of Arabidopsis *at-mmp* mutants and At2-MMP overexpressors towards the bacterial MAMP flg22. (**A**) Callose depositions detected by fluorescence microscopy. Bar = 60 μm. (**B**) Relative numbers of callose depositions. Four-week-old plants were treated with 1 μM flg22 or mock (water). At 24 h after treatment, leaves were fixed in ethanol-glacial acetic acid for several hours and stained by aniline blue for 1.5 h. Callose depositions were quantified as the number of depositions per unit of leaf surface by Image J and standardized to the mean number in WT plants. Significant differences are marked: *p < 0.05, **p < 0.01 (Student’s *t*-Test). Three independent experiments were performed with similar results.

As altered expression of At-MMPs compromised resistance to pathogens, we addressed the question whether this is due to direct proteolytic activity against the fungal pathogens or due to an endogenous function in defence signalling and activation as is proposed for mammalian MMPs [4]. We monitored a hallmark event of pattern-triggered-immunity (PTI) in Arabidopsis WT, *at-mmp* mutants and *At2-MMP*ox lines in pathogen-free system. Since callose deposition is a characteristic of PTI mediated by the leucine-rich repeat receptor kinases flagellin sensing 2 (FLS2) [29, 35–36], callose deposition was assessed in response to treatments of leaves with 1 μM of the flg22, the cognate ligand of the FLS2 receptor. Using aniline-blue staining and fluorescence microscopy, we found a regular deposition of callose by 24 h of treatment in leaves of WT plants, while mutants *at2-mmp* and *at3-mmp* showed less callose deposition, and *at2*,*3-mmp* and *at2*,*3*,*5-mmp* mutants were virtually free of callose deposition. Consistent with this, *At2-MMP*ox plants L1 and L2 showed a significantly higher level of callose deposition as compared with the WT plants (Fig 6A and 6B). These data suggest that At-MMPs are involved in MAMP-triggered defence signalling.

### Proteolytic activity of At2-MMP

**Fig 7:**
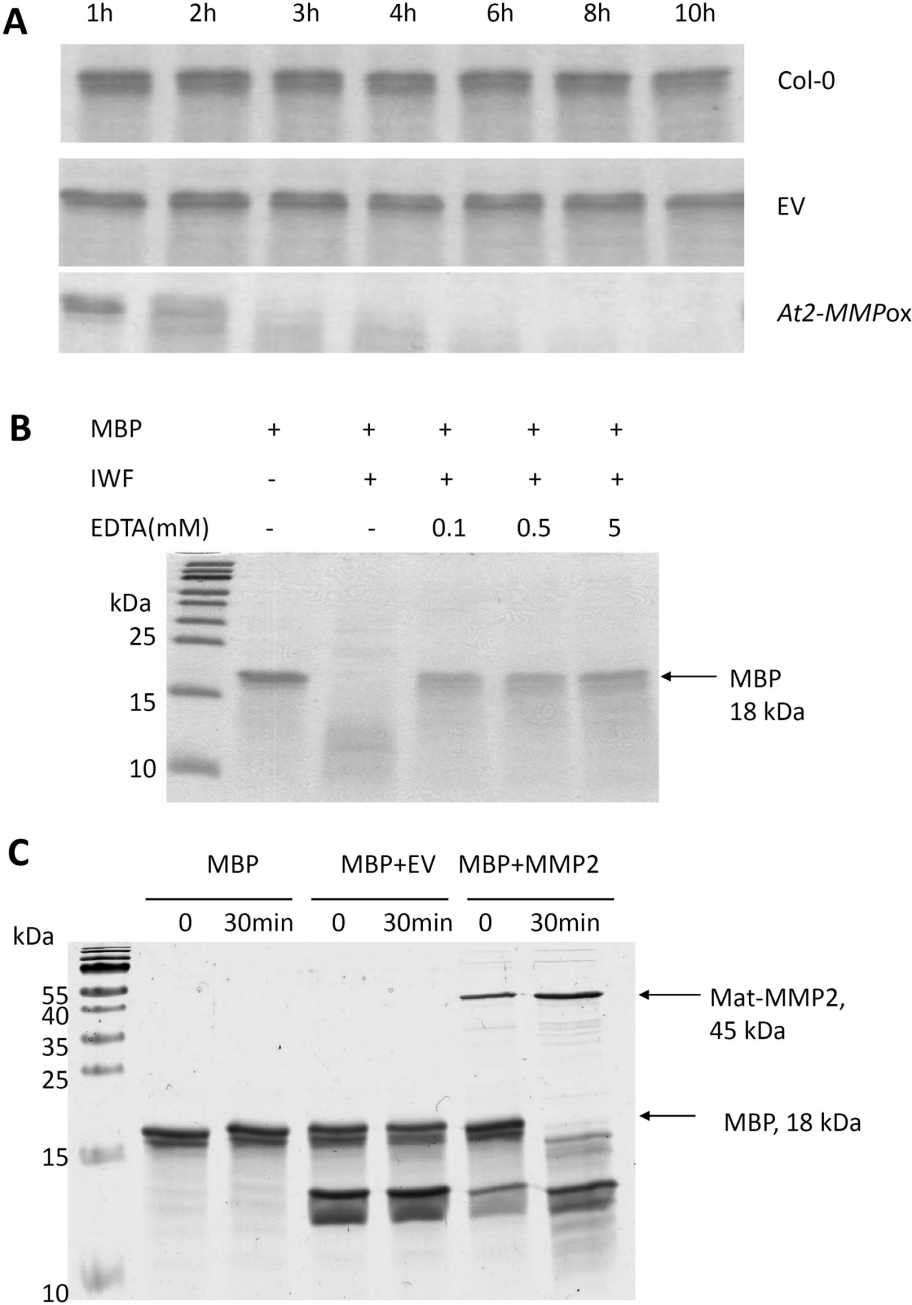
Proteolytic activity of At2-MMP. (**A**) Intercellular washing fluid (IWF) was extracted from six-week-old Arabidopsis plants using an infiltration-centrifugation method. About 30 fully-expanded rosette leaves were detached from 10 plants, IWF from transgenic and control plants were produced, with their proteolytic activities determined by degradation of myelin basic protein (MBP) and fractionation on 20% SDS-PAGE. (**B**) Activity of the IWF from At2-MMPox plants was inhibited by EDTA. (**C**) Degradation of MBP by recombinantly produced Mat-MMP2 (predicted mature At2-MMP). The Mat-MMP2 protein starts from the amino acid D in the cysteine switch motif PRCGNPD and ends before the C-terminal transmembrane domain. Both Mat-MMP2 and proteins produced from empty vector (EV) transformed cells were harvested from the supernatant after three times sonication. MBP was incubated with or without the presence of Mat-MMP2 at 37^°^C for 0 min and 30 min. Samples were separated by 20% SDS-PAGE.

Artificial substrates such as azocoll and myelin basic protein (MBP) have often been used as substrates in activity assays for plant MMPs [16, 18, 21, 29, 32]. Marino et al [37] expressed and purified the catalytic domains of the five At-MMPs as recombinant His-tag fusion proteins in *Escherichia coli*, demonstrating that all five recombinant At-MMPs exhibited proteolytic activity by degrading protease substrate. To assess the proteolytic activity of At2-MMP in the overexpression line *At2-MMP*ox L1, intercellular washing fluid (IWF) was extracted from leaves. The IWF from *At2-MMP*ox L1 and controls was incubated with MBP up to 10 h. Degradation of MBP was monitored by SDS-PAGE (Fig 7A). Under this condition, only the IWF from *At2-MMP*ox line could degrade the substrate MBP. The result confirms At2-MMP’s proteolytic activity in the apoplast. Further, we added the zinc chelator EDTA in the reaction with MBP. Consistent with the Zinc dependency of metalloproteases, the proteolytic activity of IWF from *At2-MMP*ox plants was strongly inhibited even at 0.1 mM EDTA concentrations (Fig 7B).

Callose deposition assay indicated an endogenous function of plant MMPs in defence signalling. To address the question whether At2-MMP also has a direct activity against pathogens, we produced the mature At2-MMP (Mat-MMP2), which contains only the catalytic domain as N-terminally tagged protein (S5 Fig). The recombinant protein was first tested for enzymatic activity using the substrate MBP. In the presence of Mat-MMP2 (0.1 μM), complete degradation of MBP (0.25 μg/μl) was observed within 30 min (Fig 7C). To test whether Mat-MMP2 has a direct effect on fungal spore survival and germination, *B*. *cinerea* was co-cultured with recombinant Mat-MMP2. We found that 0.1 μM Mat-MMP2 does not directly inhibit the germination and growth of the fungus compared to the vector-derived Thioredoxin-His-tag peptide (S6 Fig).

## Discussion

Several human MMPs possess a C-terminal transmembrane domain anchoring them to the plasma membrane while others are soluble localized in the matrix [6–7]. For some plant MMPs, a C-terminal transmembrane domain is predicted. Three plant MMPs, Slti114 from soybean, NtMMP1 from tobacco and Sl3-MMP from tomato, have been experimentally demonstrated to be membrane-localized using a GFP fusion approach [18–19, 38]. Due to the presence of a cleavable signal sequence at the N terminus and a predicted transmembrane domain at the C terminus (Fig 3A), At2-MMP was also supposed to be located in the plasma membrane via the transmembrane domain or the predicted GPI-anchor [21]. In line with the prediction, we observed a membrane-localized GFP signal in transiently transformed cells using an At2-MMP-GFP fusion construct. However, a diffused GFP signal was also detected in the apoplast after glycerol-induced plasmolysis but not in the cell wall. It has been suggested that leaching of a GFP signal from membrane to apoplast may occur through stress-induced phospholipase C activity that releases GPI-anchored proteins [39–40]. However, because glycerol is a mild agent that is assumed to be harmless to plasma membrane, the secretion of At2-MMP into the extracellular space is most probably due to an endogenous active release. Consistent with this, we were able to detect enhanced proteolytic activity in the IWF of *At2-MMP*ox plants without preceding plasmolysis (Fig 7A). Further experiments are required to investigate the functionality of the GPI anchor and the proposed regulation of At2-MMP release into the apoplast and activation of the proprotein.

To determine whether At-MMPs are involved in immune responses, we assessed their expression patterns in response to microbial pathogens with different life styles. Several studies indicated that some plant MMPs are pathogen responsive, while others are thought to be involved in developmental regulation [14–19]. Our study demonstrates that *At2-MMP* and *At3-MMP* are responsive to the necrotrophic fungus *B*. *cinerea* and the hemibiotrophic bacterium *Pst* (Fig 1; S1A Fig), while they were almost non-responsive to biotrophic *G*. *orontii* with the exception of *At2-MMP* (S1B Fig). It has been shown that plant *MMP* expression is regulated by plant hormones. For instance, the tomato *Sl3-MMP* showed strong response to SA and JA [19]. *NMMP1*, the first identified MMP in *Nicotiana benthamiana* was induced by ET, but not by JA [17]. On the other hand, soybean *GmMMP2* was upregulated by pathogens and yeast extracts, but not stimulated by SA or JA [16]. Likewise our results demonstrate that *B*. *cinerea*-induced expression of *At2-MMP* and *At3-MMP* does not require functional SA, JA or ET signalling (Fig 2). However, it is notable that JA treatment could stimulate *At2-MMP* expression in rosette leaves of four-week-old but not ten-week-old Arabidopsis plants [22]. Therefore, the regulatory role of JA in *At2-MMP* expression might be associated with the age of plant seedlings.

We found strong evidence for the involvement of At2-MMPs in immunity by analysing both stable transgenic overexpression lines and T-DNA insertion mutants. Constitutive overexpression of *At2-MMP* enhanced disease resistance, whereas the *at2-mmp* mutant displayed increased susceptibility to *B*. *cinerea*. Phylogenetic analysis showed that *At2-MMP*, *At3-MMP* and *At5-MMP* are close to each other and display a short genetic distance based on a sequence alignment (Fig 1B). To understand the possible functional redundancy of these At-MMPs in resistance, double and triple mutants were tested. While all the mutants showed higher susceptibility to *B*. *cinerea* and *G*. *orontii* as compared to WT plants (Fig 4, Fig 5), the triple mutant *at2*,*3*,*5-mmp* showed most severe disease symptoms, followed by *at2*,*3-mmp*. This result suggests a functional redundancy of the three *At-MMP* genes which may result from the overlapping enzyme activity.

At2-MMP is involved in developmental processes such as root growth and onset of flowering [22], indicating that the MMP acts on endogenous substrates. Additionally, a germination test using recombinant At2-MMP protein did not reveal direct antimicrobial activity on *B*. *cinerea* conidia (S6 Fig). This result hints to the possibility that the contribution of At2-MMP in defence is likely through an indirect mode of action on plant substrates. But constitutive expression of *At2-MMP* in transgenic plants did not result in the accumulation of defence marker genes, such as *PR1*, *PDF1*.*2*, and *ERF1* (S7 Fig), which also argues that At2-MMP does not act via activation of canonical defence pathways or needs to be activated during PTI. Wilson *et al* [41] demonstrated that mice matrilysin (MMP7) is involved in the innate immunity by producing active α-defensins from inactive precursors. A similar mechanism has been proposed for soybean GmMMP2, which may activate plant-derived enzymes that degrade fungal cell walls and aid in the release of antimicrobial substances [16]. Identification of MMPs’ natural substrates in plants is needed to fully understand the mechanism underlying the functioning of At2-MMP during plant immune responses. A recent study showed that subtilisin-like protease P69B is a physiologically relevant substrate of Sl2- and Sl3-MMP in tomato in a context of cell death control [20]. This is the first report about the identification of natural substrates for plant MMP proteins, thus shedding light for similar studies on other plant MMPs.

Mutations in At2-MMP resulted in late flowering and early senescence [22]. Consistent with this finding, we also observed a delayed flowering in the *at2-mmp* mutant under short-day condition (data not shown). Moreover, constitutive overexpression of *At2-MMP* resulted in early flowering clearly confirming functions of MMPs in developmental processes (S3 Fig).

To address how At-MMPs modulate plant immunity, we treated *at2-mmp*, *at3-mmp*, *at2*,*3-mmp*, and *at2*,*3*,*5-mmp* mutants as well as *At2-MMP*ox lines with the bacterial MAMP flg22. *at2*,*3*,*5-mmp* was compromised in callose deposition in comparison to WT; while highest callose deposition was observed in an *At2-MMP*ox line (Fig 6A and 6B). These observations show a requirement of At2-MMP, At3-MMP and At5-MMP for full PTI responses.

It is tempting to speculate that At2-MMP, At3-MMP and At5-MMP may be involved in host protection or production of alarming responses upon pathogen-mediated cell damage by acting on yet unidentified substrates for generation of danger signals. This hypothesis is in line with findings from insects. The immunity-related MMP from the greater wax moth *Galleria mellonella* also is induced upon bacterial and fungal infection, and enhances immune responses such as the accumulation of antimicrobial peptides [10]. Moreover, the immunity-related insect MMP degrades collagen type IV, thereby producing particular peptidic danger signals, which in turn can set the immune system into alarm [42]. The lack of collagen in plants raises the question about corresponding substrates and danger signals in plants. Their identification would help to answer the question whether the immunity-related functions of particular MMPs are evolutionary conserved or have independently been co-opted to fulfil immunity-related functions in animals and plants.

## Supporting information

S1 Fig Expression profile of *At-MMPs* in Arabidopsis (WT) leaves in response to *P. syringae* pv. *tomato* DC3000 (*Pst*) and *Golovinomyces orontii*. (**A**) Six-week-old Arabidopsis plants were infiltrated with a *Pst* suspension (OD_600_ = 0.1) in 10 mM MgCl_2_. Mock treatment was performed by infiltration with 10 mM MgCl_2_. (**B**) Expression profile of *At-MMPs* in WT leaves after *G*. *orontii* infection. Five-week-old plants were inoculated with fungal conidia by spraying a spore suspension (50×10^3^ conidia/ml in 0.005% Tween20/water). Mock treatment was performed with Tween20/water. Leaves were harvested at the indicated time points after *G*. *orontii* infection and used for total RNA extraction. RT-PCR was performed using UBQ5 as an internal control. Experiments were independently repeated three times with similar results.
(TIF)

S2 Fig Expression profile of *At-MMPs* in Arabidopsis WT roots after inoculation with the beneficial fungus *Serendipita indica*. The roots of three-week-old plants grown on ATS medium were inoculated with chlamydospores of *S*. *indica* (5×10^5^ spores/ml in 0.005% Tween20/water). The mock treatment was done with Tween20/water. Roots were harvested at the indicated time points after *S*. *indica* inoculation and used for total RNA extraction. RT-PCR was performed using UBQ5 as an internal control. The experiments were repeated two times with similar results.
(TIF)

S3 Fig Early flowering of Arabidopsis At2-MMPox plants. The phenotypes of WT, empty-vector transformant (EV), and At2-MMPox at the stage of nine weeks under short-day condition are shown.
(TIF)

S4 Fig Immune responses of the Arabidopsis *at1-mmp* mutant to *Golovinomyces orontii* infection and MAMP. Four-week-old WT plants and the *at1-mmp* mutant were spray-inoculated with 20–40×10^3^ conidia per ml spore suspension. Eleven days after inoculation, the leaves were detached to determine the amount of conidia per mg of leaf fresh weight. At least 10 individually plants were treated in each line. The bars indicate the standard error. Experiments were repeated three times with similar results.
(TIF)

S5 Fig Production of recombinant At2-MMP protein. (A), IPTG induction of recombinant At2-MMP protein in E. coli. Bacterial cells were harvested at indicated time points (0, 2 and 4 h) after addition of 1 mM IPTG and loaded on 12% SDS-PAGE. (B), Presence of recombinant At2-MMP protein in the after multiple sonications. Samples were separated on 12% SDS-PAGE.
(TIF)

S6 Fig The At2-MMP protein exhibits no direct inhibition on *Botrytis cinerea* spores. Spores were incubated with proteins produced from *E*. *coli* cells transformed with EV (pET32a (+) -empty vector) or pET32a (+)-Mat-MMP2 for 10 h at room temperature in darkness. Photograph was taken10 h after incubation.
(TIF)

S7 Fig Expression profile of *PDF1.2*, *PR1* and *ERF1*. Leaves from 6 week old Col-0, control (EV) transformant, *at2-mmp* and *At2-MMP2ox* (L1 and L2) plants were harvested at the indicated time points after *B*. *cinerea* infection and used for total RNA extraction. RT-PCR was performed using UBQ5 as an internal control.
(TIF)

S1 Table List of primers used in this study. (PDF)
