# Supplementary figures and images for: Matrix metalloproteinases operate redundantly in Arabidopsis immunity against necrotrophic and biotrophic fungal pathogens
Source: PLoS One. 2017 Aug 23;12(8):e0183577. doi: 10.1371/journal.pone.0183577 (PMC5568438; doi:10.1371/journal.pone.0183577)

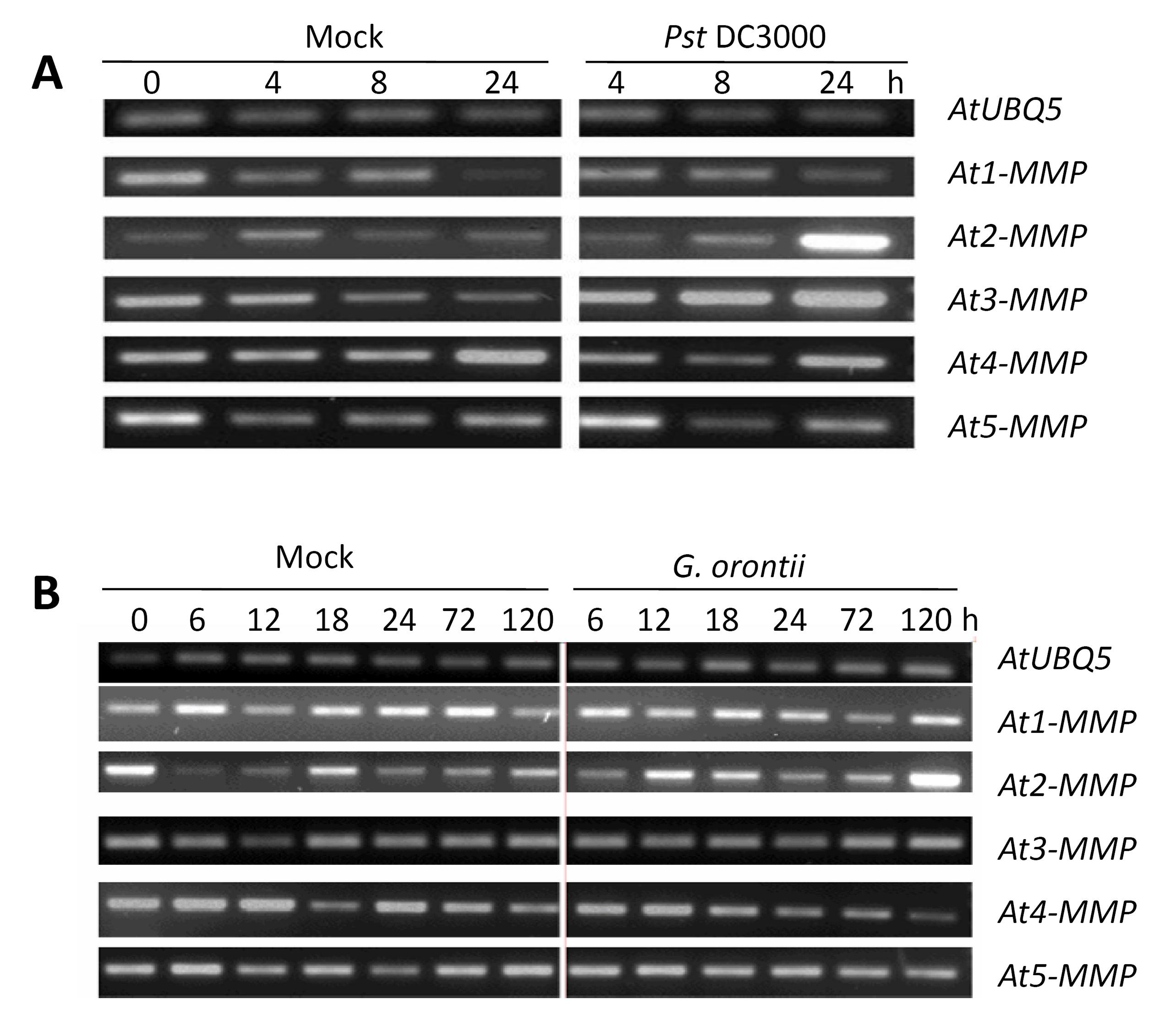

Supplement: S1 Fig — (A) Six-week-old Arabidopsis plants were infiltrated with a Pst suspension (OD600 = 0.1) in 10 mM MgCl2. Mock treatment was performed by infiltration with 10 mM MgCl2. (B) Expression profile of At-MMPs in WT leaves after G. orontii infection. Five-week-old plants were inoculated with fungal conidia by spraying a spore suspension (50×103 conidia/ml in 0.005% Tween20/water). Mock treatment was performed with Tween20/water. Leaves were harvested at the indicated time points after G. orontii infection and used for total RNA extraction. RT-PCR was performed using UBQ5 as an internal control. Experiments were independently repeated three times with similar results. (TIF) [file pone.0183577.s001.tif]

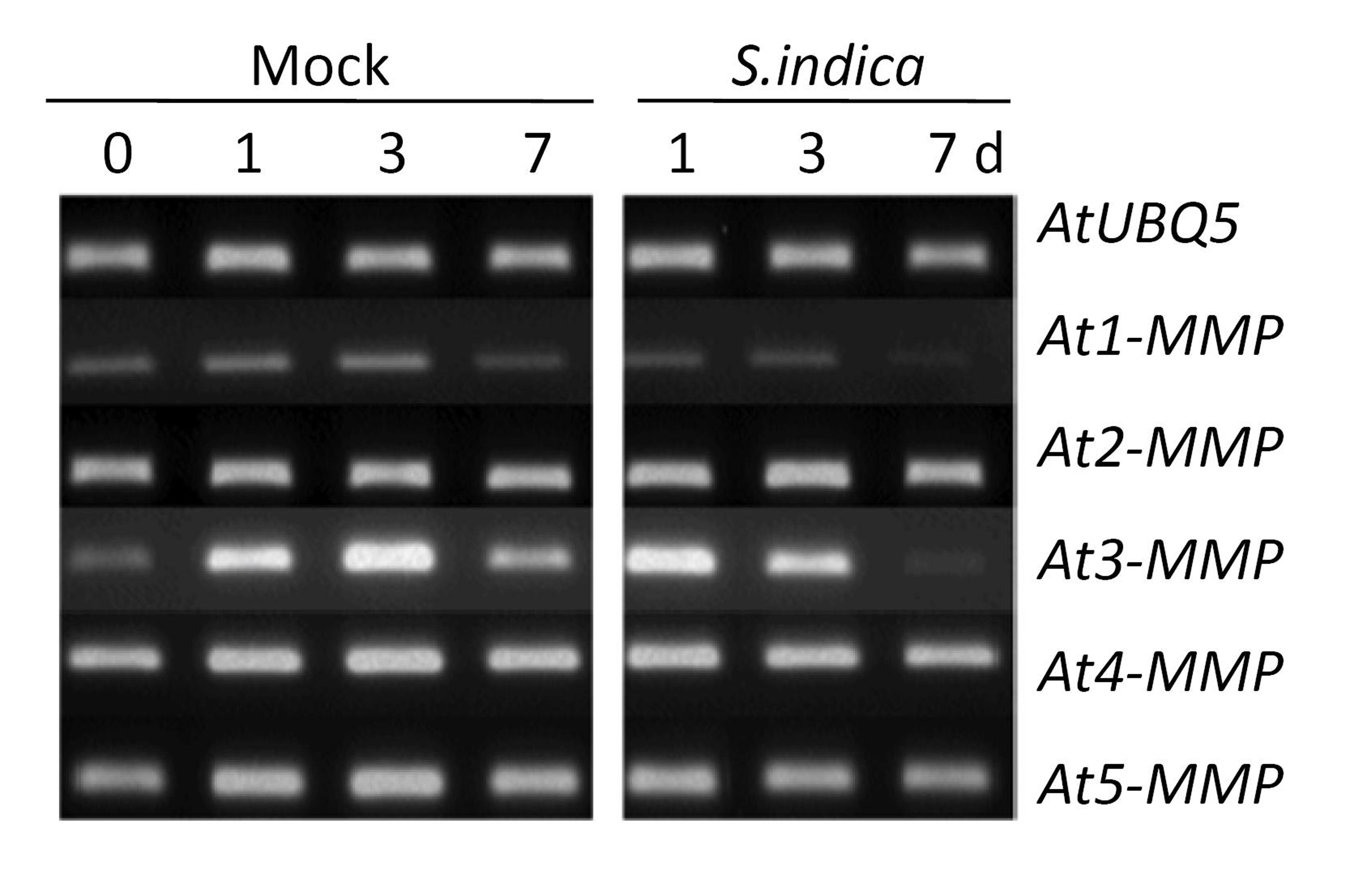

Supplement: S2 Fig — The roots of three-week-old plants grown on ATS medium were inoculated with chlamydospores of S. indica (5×10^5^ spores/ml in 0.005% Tween20/water). The mock treatment was done with Tween20/water. Roots were harvested at the indicated time points after S. indica inoculation and used for total RNA extraction. RT-PCR was performed using UBQ5 as an internal control. The experiments were repeated two times with similar results. (TIF) [file pone.0183577.s002.tif]

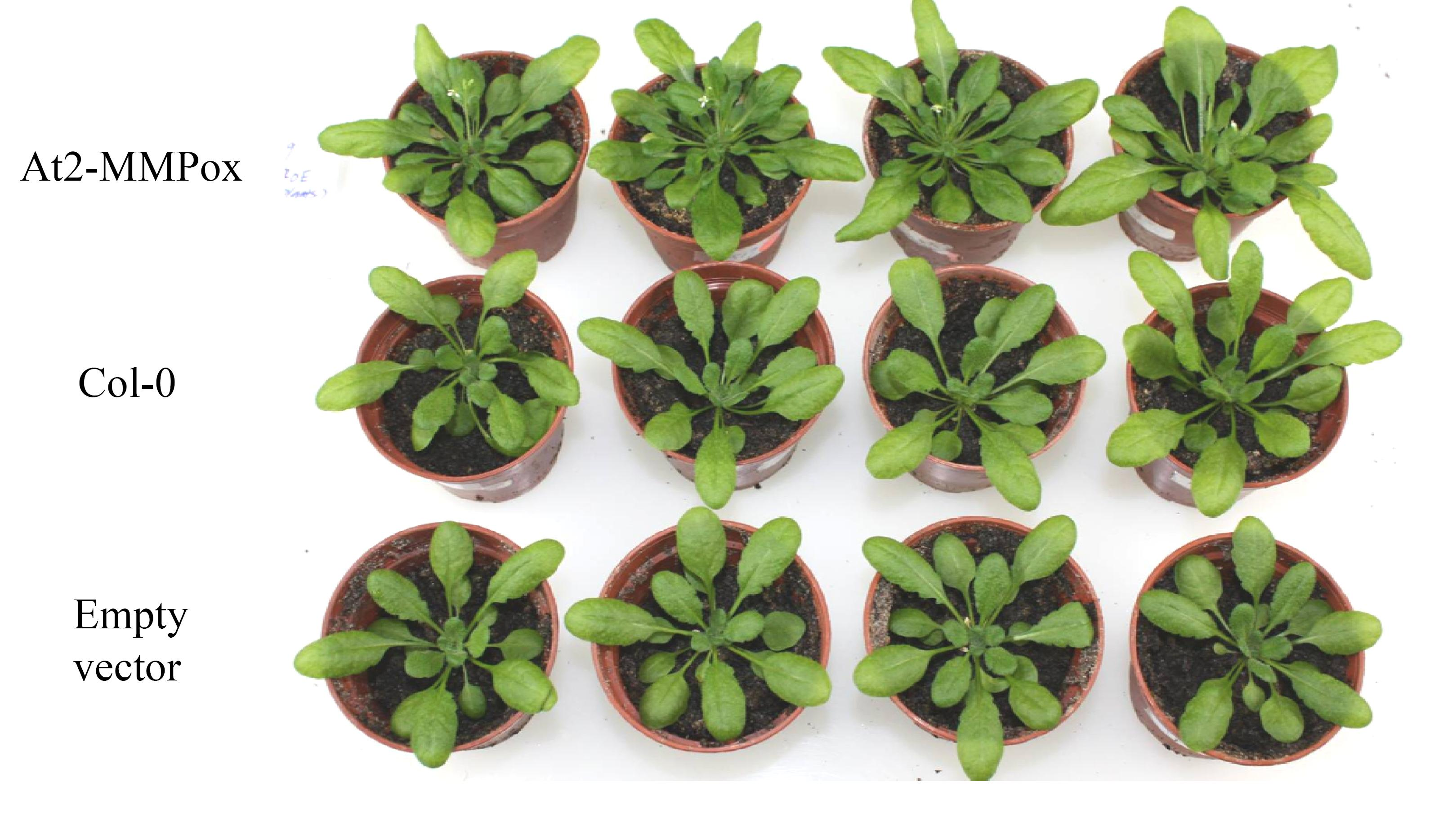

Supplement: S3 Fig — The phenotypes of WT, empty-vector transformant (EV), and At2-MMPox at the stage of nine weeks under short-day condition are shown. (TIF) [file pone.0183577.s003.tif]

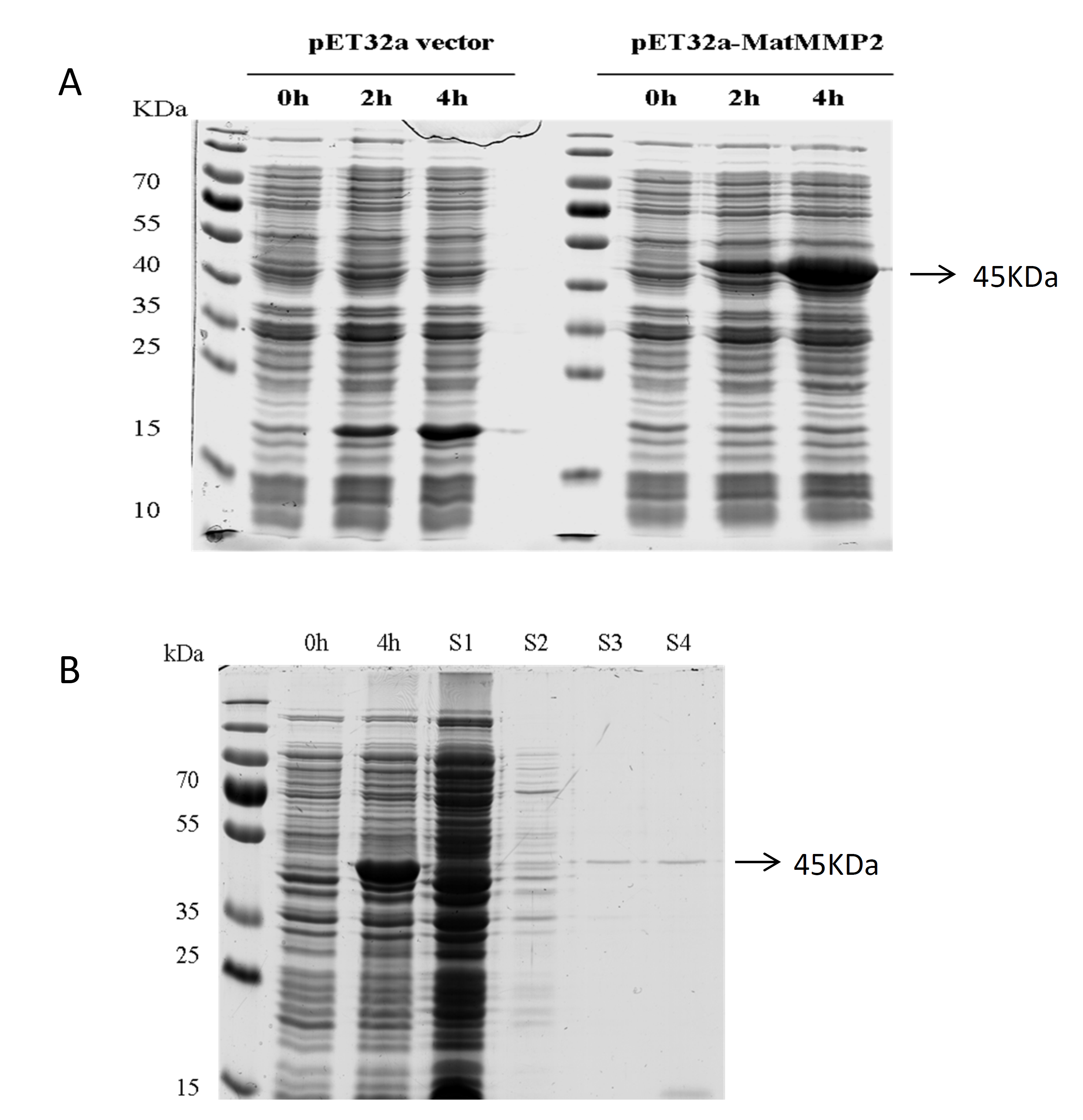

Supplement: S5 Fig — (A), IPTG induction of recombinant At2-MMP protein in E. coli. Bacterial cells were harvested at indicated time points (0, 2 and 4 h) after addition of 1 mM IPTG and loaded on 12% SDS-PAGE. (B), Presence of recombinant At2-MMP protein in the after multiple sonications. Samples were separated on 12% SDS-PAGE. (TIF) [file pone.0183577.s005.tif]

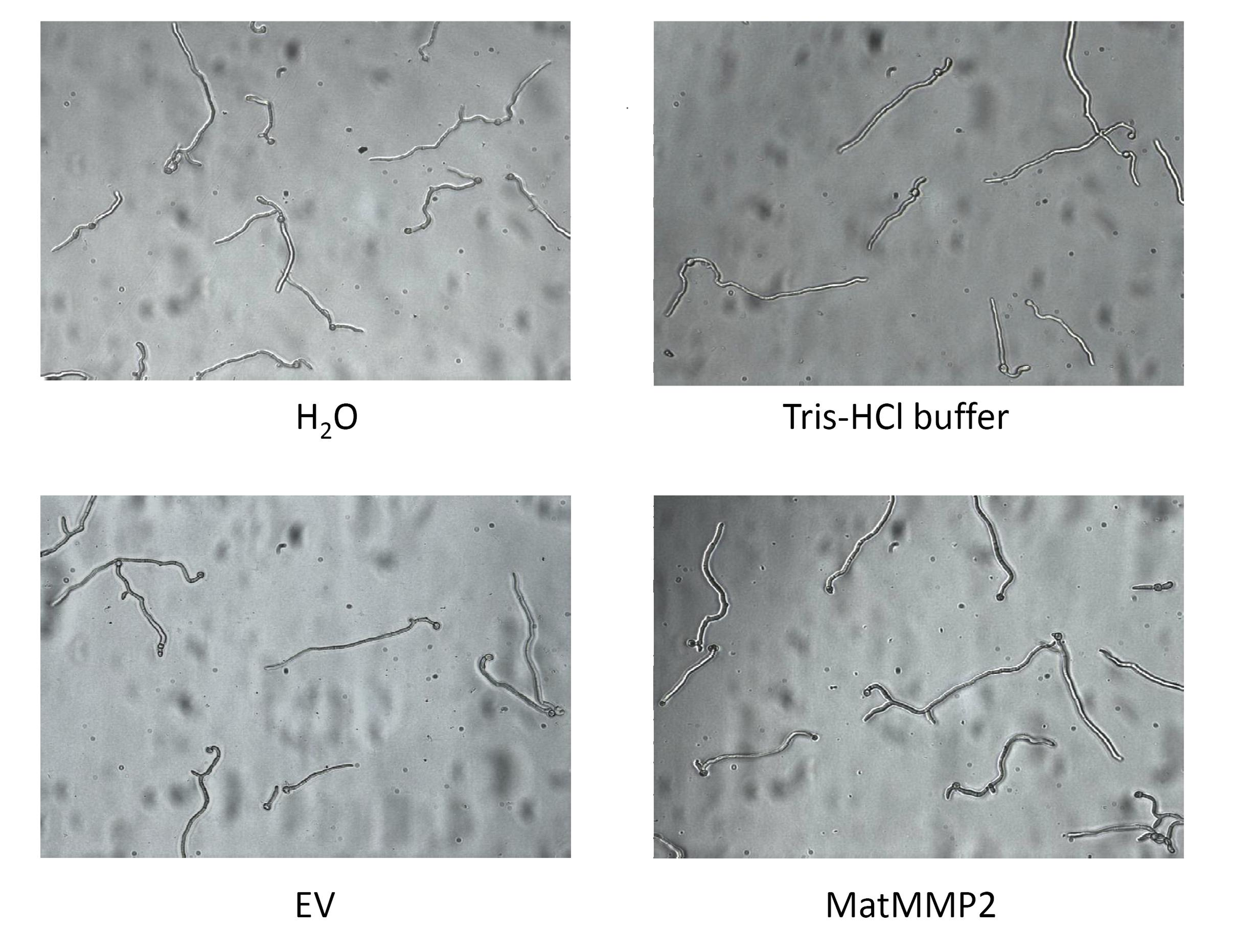

Supplement: S6 Fig — Spores were incubated with proteins produced from E. coli cells transformed with EV (pET32a (+) -empty vector) or pET32a (+)-Mat-MMP2 for 10 h at room temperature in darkness. Photograph was taken10 h after incubation. (TIF) [file pone.0183577.s006.tif]

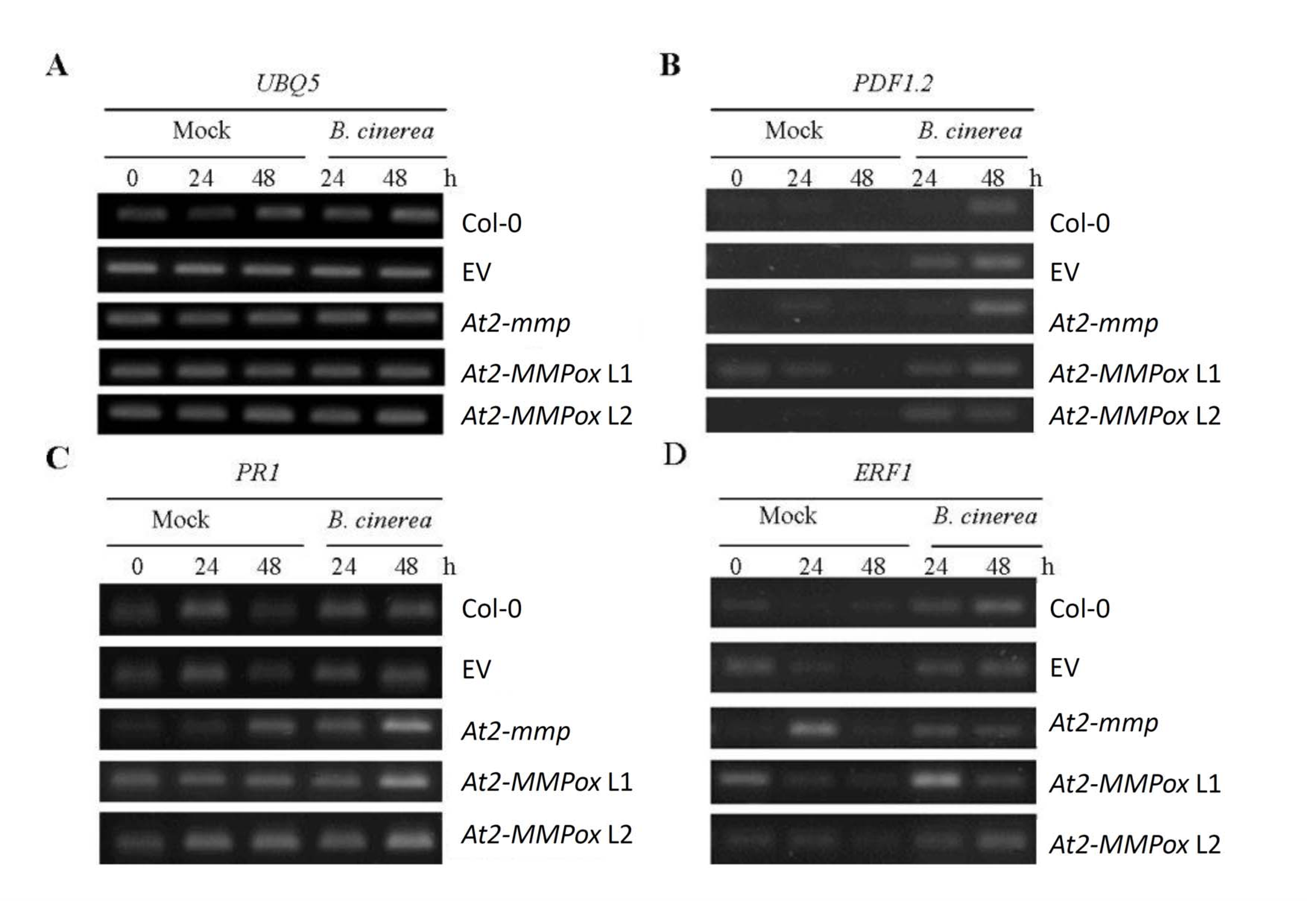

Supplement: S7 Fig — Leaves from 6 week old Col-0, control (EV) transformant, at2-mmp and At2-MMP2ox (L1 and L2) plants were harvested at the indicated time points after B. cinerea infection and used for total RNA extraction. RT-PCR was performed using UBQ5 as an internal control. (TIF) [file pone.0183577.s007.tif]
