# Supplementary material for: Matrix metalloproteinases operate redundantly in Arabidopsis immunity against necrotrophic and biotrophic fungal pathogens
Source: PLoS One. 2017 Aug 23;12(8):e0183577. doi: 10.1371/journal.pone.0183577 (PMC5568438; doi:10.1371/journal.pone.0183577)
Supplement: S1 Table — (PDF) [file pone.0183577.s008.pdf]

S1 Table. List of primers used in this study

Primer	Sequence	Use	Target
At1-MMP-F	CGATTCGGTTACGTCAATGAT	Expression analysis	*At1-MMP*
At1-MMP-R	CACGGTTCCATTTCGGTTTA	Expression analysis	*At1-MMP*
At2-MMP-F	CAACGGTACTTCGTTGATGC	Expression analysis	*At2-MMP*
At2-MMP-R	GAAGTTTAACGCGGTGACG	Expression analysis	*At2-MMP*
At3-MMP-F	GCGGTAGGAAAACCTTCGAG	Expression analysis	*At3-MMP*
At3-MMP-R	TGATGTCAGAGGTCGAGA ACC	Expression analysis	*At3-MMP*
At4-MMP-F	CGGTGATCCCAGTATCCTTC	Expression analysis	*At4-MMP*
At4-MMP-R	GCCACAGATTCCAAA TCGAC	Expression analysis	*At4-MMP*
At5-MMP-F	GCCGAA ACGGAAACGAGA	Expression analysis	*At5-MMP*
At5-MMP-R	CATGCGCTAATGTTCCCATA	Expression analysis	*At5-MMP*
AtPDF1.2-F	TGGCTTATTTCTTCACACAACACA	Expression analysis	*AtPDF1.2*
AtPDF1.2-R	AAGATTCAGTGGTCCTGTTGT	Expression analysis	*AtPDF1.2*
AtPR1-F	AGCGATGTTTACGAACCCCA	Expression analysis	*AtPR1*
AtPR1-R	CCCACGAGGATCATAGTTGCA	Expression analysis	*AtPR1*
AtERF1-F	CCATTCTCCGGCTTCTCACC	Expression analysis	*AtERF1*
AtERF1-R	TTCACGGAGCGGTGATCAAA	Expression analysis	*AtERF1*
UBQ-F	GTTAAGCTCGCTGTTCTTCAGT	Expression analysis	*AtUBQ5*
UBQ-R	TCAAGCTTCAACTCCTTCTTTC	Expression analysis	*AtUBQ5*
At2-MMP-FL-F	AT**GGATCC**AATCCGAAAACCACCATGAG	Cloning	*At2-MMP*
At2-MMP-FL-R	CA	Cloning	*At2-MMP*
GFP-SalI	CGC**GTCGAC**CATGGTGAGCAAGGGCGAGGA	Subcellular localization	*GFP*
GFP-XhoI	CCG**CTCGAG**GTCTTGTACAGCTCGTCCATGC	Subcellular localization	*GFP*
At2-MMP-XhoI	GACA	Subcellular localization	*At2-MMP*
MatMMP2-Bam5	CG**GGATCC**GACGTGGTCAACGGTACTTCG	Recombinant protein	*At2-MMP*
MatMMP2-Hind3	CCC**AAGCTT**GACCGTCGATTCTCCAGGCGGC	Recombinant protein	*At2-MMP*
N582450LP	AGAGTGAAGTTTAACGCGGTG	T-DNA mutant identification	*at2-mmp*
N582450RP	TGAATTCAATAACTCCAAATTTATAAAG	T-DNA mutant identification	*at2-mmp*
		**(to be continued)**	

**Primer**	**Sequence**	**Use**	**Target**
GABI_416E03LP	TTTCCATTGGAATCATTCACC	T-DNA mutant identification	*at2-mmp*
GABI_416E03RP	TTACGTTTCCCTGTCGTGATC	T-DNA mutant identification	*at2-mmp*
N103532_LP	TGAGTTGAGAGATCAAACCCG	T-DNA mutant identification	*at3-mmp*
N103532_RP	CGACGATATTCTCAAGAACGC	T-DNA mutant identification	*at3-mmp*
N115923_LP	GAACCTTCTACCGAGGAATGG	T-DNA mutant identification	*at3-mmp*
N115923_RP	TTTAACCGGTCCTTTACCACC	T-DNA mutant identification	*at3-mmp*
GABI_075C07LP	GTCGAATCCAAATTTGGTGTG	T-DNA mutant identification	*at4-mmp*
GABI_075C07RP	CATCGGCTAGATTTGTCGAAG	T-DNA mutant identification	*at4-mmp*
S032466.54LP	CTGCCTAATGGGCTATGTCAG	T-DNA mutant identification	*at4-mmp*
S032466.54RP	TTGGAGGTGACGTTTGATTTC	T-DNA mutant identification	*at4-mmp*
S032466.55LP	AGCCTTTTGGAAAACGATAGC	T-DNA mutant identification	*at4-mmp*
S032466.55RP	ATCTCCCGTAGCTTCTTCTCG	T-DNA mutant identification	*at4-mmp*
N619909LP	CGCCGCAGAATAATTTAACAG	T-DNA mutant identification	*at5-mmp*
N619909RP	CCTAAGAAACTACCATTAATGATGCTC	T-DNA mutant identification	*at5-mmp*
N593137LP	CGCCGCAGAATAATTTAACAG	T-DNA mutant identification	*at5-mmp*
N593137RP	TGTTGTATGTGATGACTTCGTAAGTC	T-DNA mutant identification	*at5-mmp*
N656052LP	AAA TCG TCA GTG CAA TTA CCG	T-DNA mutant identification	*at5-mmp*
N656052RP	TCG AAC TAA GCC GAG TGA AAC	T-DNA mutant identification	*at5-mmp*
N660426LP	ATGTTCACCGGAGAAAAATCC	T-DNA mutant identification	*at5-mmp*
N660426RP	TCAGAAAATGGTTTCTCTCGG	T-DNA mutant identification	*at5-mmp*
Spm32	TACGAATAAGAGCGTCCATTTTAGAGTGA	T-DNA mutant identification	T-DNA border
LBb1	GCGTGGACCGCTTGCTGCAACT	T-DNA mutant identification	T-DNA border
GABI_LB	CCCATTTGGACGTGAATGTAGACAC	T-DNA mutant identification	T-DNA border
GABI_RB	CCAAAGATGGACCCCCACCCAC	T-DNA mutant identification	T-DNA border

Note:introduced restriction sites are in bold fonts
